# A Novel Approach to Cadaver‐Based Soiled Airway Simulation

**DOI:** 10.1002/aet2.70182

**Published:** 2026-05-11

**Authors:** Adam Parks, Sean Hurley, Patricia Marks, James Gould

**Affiliations:** ^1^ Department of Emergency Medicine Dalhousie University Halifax Nova Scotia Canada; ^2^ Department of Emergency Medicine University of British Columbia Vancouver British Columbia Canada

**Keywords:** airway contamination, airway management, simulation training, soiled airway

## Abstract

Effective management of heavily soiled airways is a critical and challenging skill across multiple clinical settings. Existing training methods rely largely on manikin‐based simulators, which often lack sufficient anatomical realism and haptic fidelity. Here, we describe a novel, low‐cost, and practical technique for simulating soiled airways in clinical‐grade cadavers. The method employs readily available equipment, including a bougie, Foley catheter, and continuous bladder irrigation system, to rapidly deliver simulated bloody emesis into the proximal esophagus and oropharynx. This technique was successfully implemented on nine clinical‐grade cadavers during emergency medicine (EM) and anesthesiology physician airway training sessions. All surveyed instructors reported marked improvements in realism and haptic fidelity compared with traditional manikin‐based approaches, and no adverse events occurred during cadaver preparation or course delivery. This novel, cadaver‐based soiled airway simulation technique offers a practical and reproducible approach to enhance soiled airway education and research.

## 1 Introduction

Training for the effective and efficient management of airway soiling is important across multiple disciplines, including prehospital and emergency medicine (EM), critical care, and anesthesiology. Techniques to manage soiled airways have been well‐described and require specific training to be utilized effectively [1, 2]. While various training methods and simulators have emerged to prepare providers for these scenarios, but most rely on low‐fidelity, manikin‐based task trainers [3]. Although manikins play a foundational role in medical education, they often lack the realism and haptic feedback required to effectively simulate complex, soiled airway situations [4]. Conversely, clinical‐grade cadavers offer superior anatomical and haptic fidelity, better replicating the experience of intubating a living, anesthetized patient [5]. Given this, the development of a practical and simplistic method of simulating airway soiling in clinical‐grade cadavers may allow for airway educators and researchers to improve upon current educational and clinical techniques.

## 2 Methods

Our primary objective was to design and describe a novel, simplistic cadaver‐based soiled airway simulation technique using clinical‐grade cadavers supplied by the Dalhousie Human Body Donation Program in Halifax, Nova Scotia, Canada. Following development, we piloted airway soiling simulation during EM and anesthesiology staff physician, cadaver‐based, resuscitative procedural skills courses. Following these courses, study personnel were surveyed regarding whether the cadaver‐based technique improved upon existing manikin‐based techniques with respect to realism and haptic fidelity. Research ethics board exemption (NSH REB #1031571) was obtained for our study. Approval for the use of cadaver images in our publication was obtained from the Dalhousie University Department of Medical Neuroscience Clinical Cadaver Program.

## 3 Results

**FIGURE 1:**
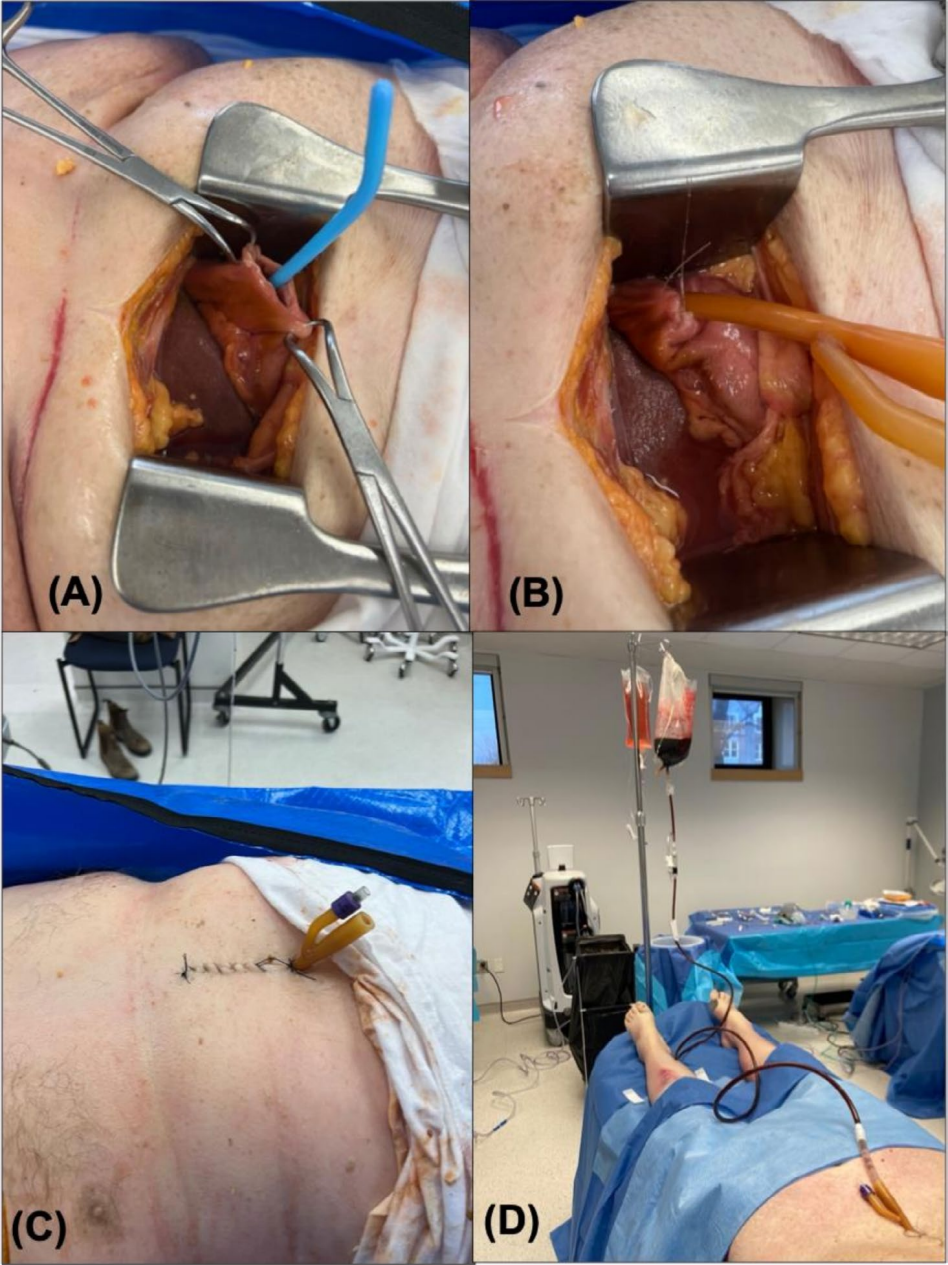
Stepwise approach to cadaver‐based soiled airway simulation. (A) A bougie is placed in the esophagus and advanced into the stomach. A small midline laparotomy incision is then made just below the xiphoid process. With the bougie in place, stomach tissue is tented and easily identifiable, and a small gastrotomy incision is made. (B) A Foley catheter is then loaded onto the bougie and pulled back into the proximal esophagus. (C) With the Foley positioned in the proximal esophagus, the balloon is inflated, the bougie is removed, and the incisions are closed. (D) The Foley catheter is then attached to standard bladder irrigation bags filled with simulated emesis. The irrigation line's manual flow control can be used to control the flow of simulated emesis.

Our novel, simplistic technique—delineated in Figure 1—was successfully utilized on nine clinical‐grade cadavers during both EM and anesthesiology staff physician training sessions. Equipment utilized for this technique is inexpensive (less than $100 USD) and readily available: a bougie, Foley catheter (size 20‐22F), and a standard continuous bladder irrigation system (including a 3‐L irrigation bag). Irrigation bags are filled with simulated bloody emesis, consisting of water, food coloring (red and black), and a thickening agent (e.g., xantham gum). During training courses, the bladder irrigation system was able to deliver up to approximately 30 mL of simulated bloody emesis per second (1.8 L per minute). Once irrigation bag(s) are empty, the simulated emesis can be recycled, by pouring suction canister contents back into an opening at the top of the irrigation bag.

Six study personnel were surveyed and all strongly agreed that this novel technique improved upon the realism and haptic fidelity of existing manikin‐based techniques. There were no adverse events and/or complications during either cadaver preparation or training courses.

## 4 Discussion

Our novel technique has proven to be practical, cost‐effective, and effective in improving the fidelity and realism of existing manikin‐based techniques. To the best of our knowledge, there has been only one previous publication describing cadaver‐based soiled airway simulation, which required a relatively invasive and complex surgical approach, including a thoracotomy and mediastinal dissection to simulate gastrointestinal contamination [6]. Given our technique's simplistic and minimally invasive nature, it can likely be employed more broadly, as providers do not require extensive surgical knowledge.

Our protocol has a number of limitations, including that it is primarily descriptive in nature and that it employs clinical‐grade cadavers, which are not readily available for the majority of clinicians and educators. The scarce nature of clinical‐grade cadavers means that every effort should be taken to optimize cadaver‐based education and research.

Utilizing this novel approach, we may not only improve upon the realism and fidelity of existing soiled airway training protocols, but also pursue performance‐based research to develop and optimize novel airway devices, management strategies, and training methodologies to improve soiled airway management.

## Author Contributions

A.P., S.H., P.M., and J.G. conceived and designed the technique. A.P. obtained research funding and drafted the manuscript, and all authors contributed substantially to its revision.

## Funding

This study was supported through the QEII Foundation's EM Young Researcher's Grant program (Halifax, Nova Scotia, Canada).

## Consent

The authors affirm that approval has been obtained from the Dalhousie University Department of Medical Neuroscience Clinical Cadaver Program for publication of the images in Figure 1.

## Conflicts of Interest

A.P., S.H., P.M., and J.G. have all received honoraria for teaching national‐level airway and resuscitative procedural courses, including AIME (Airway Interventions & Management in Emergencies) and The Resus Course.

## Data Availability

The entire detailed study protocol is available upon request, from the date of article publication by contacting Adam Parks MD, FRCPC, at a.parks@dal.ca.
